# Effect of Proteinuria Before Lenvatinib Administration on Treatment Response After Atezolizumab Bevacizumab Combination Therapy

**DOI:** 10.1002/jgh3.70098

**Published:** 2025-01-19

**Authors:** Hironori Ochi, Masayuki Kurosaki, Takaaki Tanaka, Nobuharu Tamaki, Kaoru Tsuchiya, Yutaka Yasui, Hiroyuki Marusawa, Toshifumi Tada, Shinichiro Nakamura, Takehiro Akahane, Eisuke Okamoto, Haruhiko Kobashi, Hirotaka Arai, Michiko Nonogi, Namiki Izumi

**Affiliations:** ^1^ Center for Liver‐Biliary‐Pancreatic Disease, Matsuyama Red Cross Hospital Ehime Japan; ^2^ Department of Gastroenterology and Hepatology Musashino Red Cross Hospital Tokyo Japan; ^3^ Department of Gastroenterology and Hepatology Osaka Red Cross Hospital Osaka Japan; ^4^ Department of Internal Medicine Himeji Red Cross Hospital Hyogo Japan; ^5^ Department of Gastroenterology Ishinomaki Red Cross Hospital Miyagi Japan; ^6^ Department of Gastroenterology Masuda Red Cross Hospital Shimane Japan; ^7^ Department of Gastroenterology Japanese Red Cross Okayama Hospital Okayama Japan; ^8^ Department of Gastroenterology Maebashi Red Cross Hospital Gunma Japan; ^9^ Department of Gastroenterology Tokushima Red Cross Hospital Tokushima Japan

**Keywords:** atezolizumab‐bevacizumab, hepatocellular carcinoma, lenvatinib, proteinuria

## Abstract

**Aim:**

We investigated the impact of proteinuria on the therapeutic effect before lenvatinib administration as second‐line treatment after atezolizumab‐bevacizumab.

**Methods:**

We examined 64 patients who were administered lenvatinib as second‐line treatment after discontinuation of atezolizumab and bevacizumab. Proteinuria assessed before lenvatinib administration was considered severe if the qualitative value test (QV) was 3+ or the urine protein/creatinine ratio (UPCR) was ≥ 2.0 (group A, *n* = 13) and non‐severe if the UPCR was < 2.0 or the QV was ≤ 2+ (group B, *n* = 51).

**Results:**

In the entire cohort, the modified albumin–bilirubin grades were grades 1, 2a, 2b, and 3 in 12, 21, 26, and 5 patients, respectively. Regarding the Barcelona Clinic of Liver Cancer stage, 2, 22, and 40 patients had stages A, B, and C, respectively. The objective response rate (ORR) was 14.0% and the disease control rate (DCR) was 59.3%. The median survival time and progression free survival after administration of lenvatinib was 14.8 (95% confidence interval [CI], 11.3–18.4) and 5.5 (95% CI, 3.6–7.5) months, respectively. The ORR and DCR were 0% and 38.4% for group A (*n* = 13) and 17.6% and 64.7% for group B (*n* = 51), respectively. The median time to treatment failure was 2.2 months in group A and 4.2 months in group B (*p* = 0.120).

**Conclusions:**

Severe proteinuria before lenvatinib as a second‐line therapy after atezolizumab‐bevacizumab treatment may affect the duration of lenvatinib administration and treatment efficacy.

**Abbreviations**
HCC: hepatocellular carcinoma
LEN: lenvatinib
MTA: multi‐molecular target agent
VEGF: vascular endothelial 4 growth factor

## 1 Introduction

Hepatocellular carcinoma (HCC) is the third leading cause of cancer‐related death worldwide [1]. The first‐line agents for systemic therapy, atezolizumab, an immune checkpoint inhibitor, and bevacizumab, a vascular endothelial growth factor (VEGF) inhibitor, are recommended for combination therapy (AB treatment).

However, there is no fixed second‐line treatment after the discontinuation of AB treatment, and this remains controversial. There are only few reports on lenvatinib (LEN) as second‐line therapy after AB treatment [2].

Proteinuria is an adverse event (AE) associated with AB treatment, but it is also a common AE associated with multi‐molecular target agent (MTA), including LEN. Although some AEs have been associated with the effectiveness of AB treatment [3], there are no reports on whether proteinuria after AB treatment affects the therapeutic effects of LEN.

Therefore, in this study, we aimed to analyze the treatment efficacy and survival of LEN as a second‐line therapy after AB treatment, as well as the therapeutic effect of proteinuria on LEN, using retrospective and multicenter data.

## 2 Methods

In this retrospective study, data of all patients who received AB treatment as first‐line treatment for unresectable HCCs in institutions belonging to the Japanese Red Cross Liver Study Group from September 2020 to June 2023 were collected. Patients who received LEN as a second‐line treatment for discontinued treatment due to progression disease (PD) or AEs after starting AB treatment were included in the analysis. HCC diagnosis was either pathological or imaging examination according to the guidelines of American Association for the Study of Liver Diseases and the Japan Society of Hepatology [4].

The choice of treatment method after AB treatment for the second‐line was then determined by each attending physician. LEN was administered orally: 8 mg if the patient weighed < 60 kg and 12 mg if the patient weighed ≥ 60 kg. However, some patients were started on a reduced dose at the discretion of the attending physician, considering the hepatic reserve and other factors prior to the administration of LEN. If a patient was clinically diagnosed with PD or had a serious AE after treatment initiation, LEN was discontinued at the discretion of the attending physician. Laboratory data and imaging studies prior to LEN administration were collected from electronic medical records. The albumin–bilirubin (ALBI) score and modified ALBI grade were defined according to previously published reports [5, 6].

Proteinuria was evaluated by the urine protein/creatinine ratio (UPCR); in cases where UPCR was not available, a qualitative value test (QV) using a urine dipstick was performed. Severe proteinuria was considered if QV of 3+ or UPCR ≥ 2.0 (Group A) and non‐severe if UPCR < 2.0 or QV of ≤ 2 (Group B).

Dynamic computed tomography or magnetic resonance imaging was performed every 1–3 months after initiating treatment to determine treatment efficacy according to RECIST ver1.1. AEs were evaluated using the National Cancer Institute Common Terminology Criteria for Adverse Events version 5.0.

Categorical and continuous variables of both groups were analyzed using the Mann–Whitney U test and chi‐square test, as appropriate. Overall survival (OS) and progression‐free survival (PFS) were estimated using the Kaplan–Meier method. The time from the start of treatment to the discontinuation of treatment for any reason including adverse events, progression of disease or death was defined as the time to treatment failure (TTF). Survival and disease progression rates were estimated using the Kaplan–Meier method, and differences in TTF were assessed using the log‐rank test.

In all statistical analyses, a *p*‐value of < 0.05 was considered statistically significant. All statistical analyses were performed using SPSS ver.29.

## 3 Results

### 3.1 Baseline Characteristics

Overall, 64 patients received LEN as a second‐line treatment after AB treatment discontinuation. Clinical and demographic data before LEN administration are presented in Table S1 (*n* = 64). A total of 50 patients discontinued due to PD and 12 due to AEs. AEs included fatigue in three patients, proteinuria in two, fever in two, skin rash in two, and other reasons in three patients.

The initial dose of LEN was 12 mg in 14 patients, 8 mg in 44 patients, and 4 mg in 6 patients. While 22 patients started on a dose lower than the weight‐based standard dosage.

The objective response rate (ORR) of LEN was 14.0% and disease control rate (DCR) was 59.3%. The median OS after LEN administration was 14.8 months (95% confidence interval [CI], 11.3–18.4; Figure S1a). The median PFS after LEN administration was 5.5 months, respectively (95% CI, 3.6–7.5; Figure S1b).

The median time from the discontinuation of AB treatment to LEN administration was 29 days. Severe proteinuria prior to LEN administration was observed in 13 patients (Group A), and no severe proteinuria was observed in 51 patients (Group B). The baseline characteristics of the two groups are shown in Table S2. There were no significant differences in these characteristics between the two groups. The ORR and DCR were 0% and 38.4% for Group A and 17.6% and 64.7% for Group B, respectively (Table S3).

The response rate of LEN was 13.3% (two patients) for AB treatment responders and 14.8% (seven patients) for non‐responders (*p* = 0.750). No difference in median duration of AB treatment between LEN responders (1.8 months [interquartile range 0.9–8.2]) and non‐responders (4.8 months [interquartile range 1.8–8.7]) (*p* = 0.121) was observed. The ORR for LEN was 18% for PD cases and 0% for AE cases (*p* = 0.45). Therefore, there was no significant effect of response to first‐line AB treatment, duration of treatment, or reason for discontinuation on the response to treatment with LEN.

**FIGURE 1:**
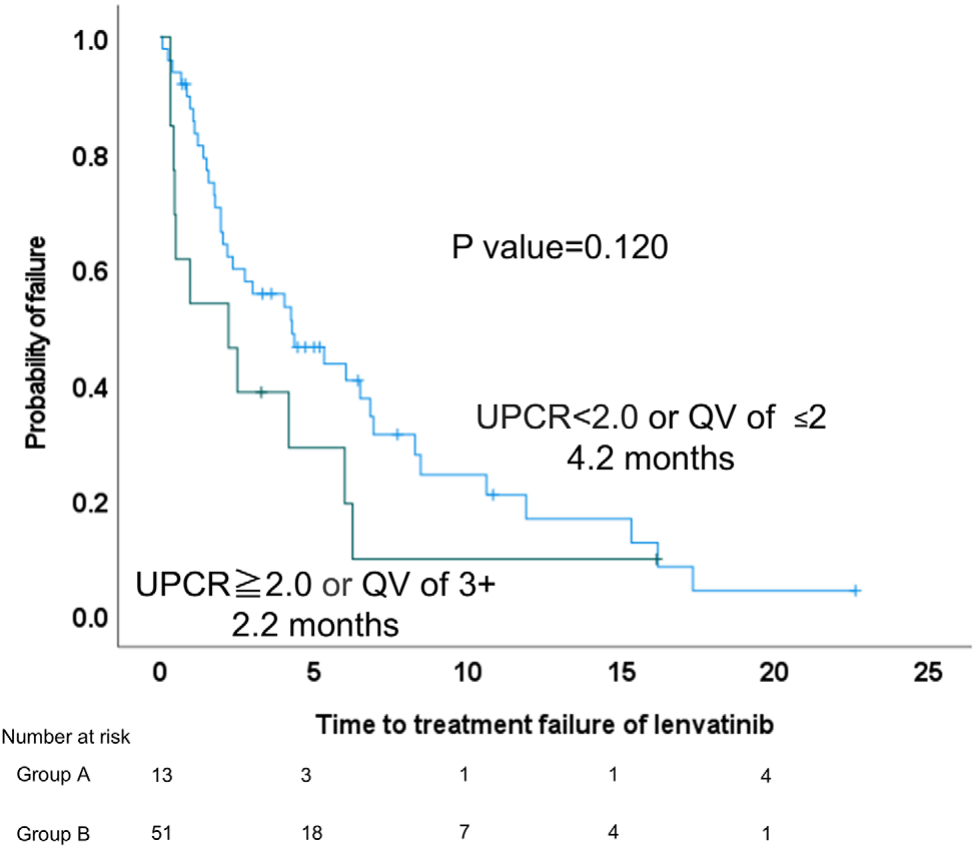
Median time to treatment failure was 2.2 months for Group A and 4.2 months for Group B (*p* = 0.120). Severe proteinuria was considered if QV of 3+ or UPCR ≥ 2.0 (Group A) and non‐severe if UPCR < 2.0 or QV of ≤ 2 (Group B). UPCR, urine protein/creatinine ratio; QV, qualitative value test.

The median TTF was 2.2 months in Group A and 4.2 months in Group B, with a trend toward a longer TTF in Group A (*p* = 0.120) (Figure 1). There were 11 discontinuations of LEN in Group A and 37 in Group B during the observation period. The percentage of AEs in the discontinued cases was 54.5% in Group A and 48.6% in Group B, with no significant difference (*p* = 0.604).

## 4 Discussion

There have been no reports on the differences in treatment effects due to proteinuria prior to LEN administration as a second‐line, and to the best of our knowledge, this is the first of such reports. We found that Group B had better disease control.

In this study, severe proteinuria was defined qualitatively using urine dipstick tests or UPCR. In this study, the cutoff value for UPCR was set at 2.0 because bevacizumab withdrawal is recommended in patients with UPCR ≥ 2.0 for AB treatment. Since the UPCR could not be measured in all cases, a QV ≥ 3 was evaluated as equivalent to a UPCR ≥ 2 for qualitative testing. This was set based on a report by Nakamura et al. showing that the UPCR was < 2.0 in 234 of 269 cases with a QV of 2 [7]. The reason we have established this criterion is because the criteria for withdrawal of bevacizumab in IMbrave0150 states “If 24‐hour proteinuria > 2 g, hold bevacizumab treatment.” In actual clinical practice in Japan, 24‐h urine collection is difficult, so UPCR is used as an alternative indicator.

Proteinuria during systemic therapy is an important AE, especially when drugs with anti‐VEGF inhibitors are used. Renal glomerular filtration membranes are composed of endothelial cells, basement membranes, and octopodial cells. The interaction between VEGF produced by octopoids and VEGFR‐2 in glomerular endothelial cells is essential for normal glomerular filtration function and repair. Inhibitors of VEGF, such as bevacizumab or LEN, are thought to disrupt glomerular structure and filtration function, resulting in urinary proteins [8].

The occurrence of proteinuria after initiation of LEN as the first‐line in the REFLECT trial was reported to improve prognosis after treatment initiation [9]. This was thought to be due to the anti‐VEGF effect exerted by the occurrence of proteinuria, which may represent the therapeutic effect of VEGF inhibition. However, as in the present study, pre‐treatment proteinuria may affect the course of treatment, and Mizuno et al. reported that the presence of pre‐treatment proteinuria in patients treated with LEN affected prognosis [10]. In this study, Group B tended to have better DCR and longer TTF. TTF did not differ significantly between the two groups in this study, but the TTF over the entire period is higher for Group B than Group A. Since there was no difference in discontinuation rates due to AEs between the two groups, it was thought that less pre‐treatment proteinuria would result in better disease control and a longer time to discontinuation of treatment. In this study, RDI was not significantly different between the two groups; the association between RDI of LEN in second‐line therapy and disease control needs to be studied in a larger number of cases, as there are few reported cases.

This study has some limitations. First, it is a retrospective study; therefore, there is a selection bias in cases where LEN was administered as second‐line therapy. Second, the number of cases is small and the observation period is short. Third, the duration of AB treatment was shorter in Group B.

In conclusion, proteinuria prior to LEN as a second‐line therapy after AB treatment may affect the duration of LEN administration and treatment efficacy.

## Ethics Statement

This research has been approved by the authors' institutional review board or equivalent committee. This research was conducted in accordance with the principles of the Declaration of Helsinki.

## Consent

Informed consent was obtained in the form of opt‐out on the website.

## Conflicts of Interest

MK, NI, and KT received lecture fees from Chugai. The other authors declare no conflicts of interest.

## Supporting information


**FIGURE S1.** (a) Median overall survival time after LEN was 14.8 months (95% confidence interval [CI], 11.3–18.4). (b) Median progression‐free survival time after LEN administration was 5.5 months (95% CI, 3.6–7.5). LEN, lenvatinib.

**TABLE S1.** Baseline patient characteristics.

**TABLE S2.** Baseline patient characteristics in each group.

**TABLE S3.** Treatment best response according to urinary protein level prior to lenvatinib administration.

## References

[1] H. Sung , J. Ferlay , R. L. Siegel , et al., “Global Cancer Statistics 2020: GLOBOCAN Estimates of Incidence and Mortality Worldwide for 36 Cancers in 185 Countries,” CA: A Cancer Journal for Clinicians 71 (2021): 209–249. 33538338 10.3322/caac.21660

[2] H. Muto , T. Kuzuya , N. Kawabe , et al., “Clinical Outcomes With Lenvatinib in Patients Previously Treated With Atezolizumab/Bevacizumab for Advanced Hepatocellular Carcinoma,” Anticancer Research 43 (2023): 4673–4682. 37772587 10.21873/anticanres.16663

[3] S. Takaki , M. Kurosaki , N. Mori , et al., “Effects on Survival of the Adverse Event of Atezolizumab Plus Bevacizumab for Hepatocellular Carcinoma: A Multicenter Study by the Japan Red Cross Liver Study Group,” Investigational New Drugs 41 (2023): 340–349. 36995548 10.1007/s10637-023-01349-4

[4] N. Kokudo , N. Takemura , K. Hasegawa , et al., “Clinical Practice Guidelines for Hepatocellular Carcinoma: The Japan Society of Hepatology 2017 (4th JSH‐HCC Guidelines) 2019 Update,” Hepatology Research 49 (2019): 1109–1113. 31336394 10.1111/hepr.13411

[5] A. Hiraoka , K. Michitaka , T. Kumada , et al., “Validation and Potential of Albumin‐Bilirubin Grade and Prognostication in a Nationwide Survey of 46,681 Hepatocellular Carcinoma Patients in Japan: The Need for a More Detailed Evaluation of Hepatic Function,” Liver Cancer 6 (2017): 325–336. 29234636 10.1159/000479984 PMC5704689

[6] A. Hiraoka , K. Michitaka , T. Kumada , and M. Kudo , “ALBI Score as a Novel Tool in Staging and Treatment Planning for Hepatocellular Carcinoma: Advantage of ALBI Grade for Universal Assessment of Hepatic Function,” Liver Cancer 6 (2017): 377–379. 29234641 10.1159/000481212 PMC5704723

[7] M. Nakamura , T. Funakoshi , S. Kataoka , et al., “Decision Making for Anti‐VEGF Inhibitor Continuation: Dip Stick? Or Urine Protein/Creatinine Ratio? (VERSiON UP Study),” BMC Cancer 22 (2022): 515. 35525917 10.1186/s12885-022-09611-3 PMC9080145

[8] V. Eremina , J. A. Jefferson , J. Kowalewska , et al., “VEGF Inhibition and Renal Thrombotic Microangiopathy,” New England Journal of Medicine 358 (2008): 1129–1136. 18337603 10.1056/NEJMoa0707330 PMC3030578

[9] M. W. Sung , R. S. Finn , S. Qin , et al., “Association Between Overall Survival and Adverse Events With LEN Treatment in Patients With Hepatocellular Carcinoma (REFLECT),” Journal of Clinical Oncology 37 (2019): 317.

[10] K. Mizuno , N. Imai , T. Yamamoto , et al., “Pretreatment Proteinuria Predicts the Prognosis of Patients Receiving Systemic Therapy for Unresectable Hepatocellular Carcinoma,” Cancers (Basel) 15 (2023): 2853. 37345189 10.3390/cancers15102853 PMC10216745

