# Supplementary figures and images for: Effect of Proteinuria Before Lenvatinib Administration on Treatment Response After Atezolizumab Bevacizumab Combination Therapy
Source: JGH Open. 2025 Jan 19;9(1):e70098. doi: 10.1002/jgh3.70098 (PMC11743979; doi:10.1002/jgh3.70098)

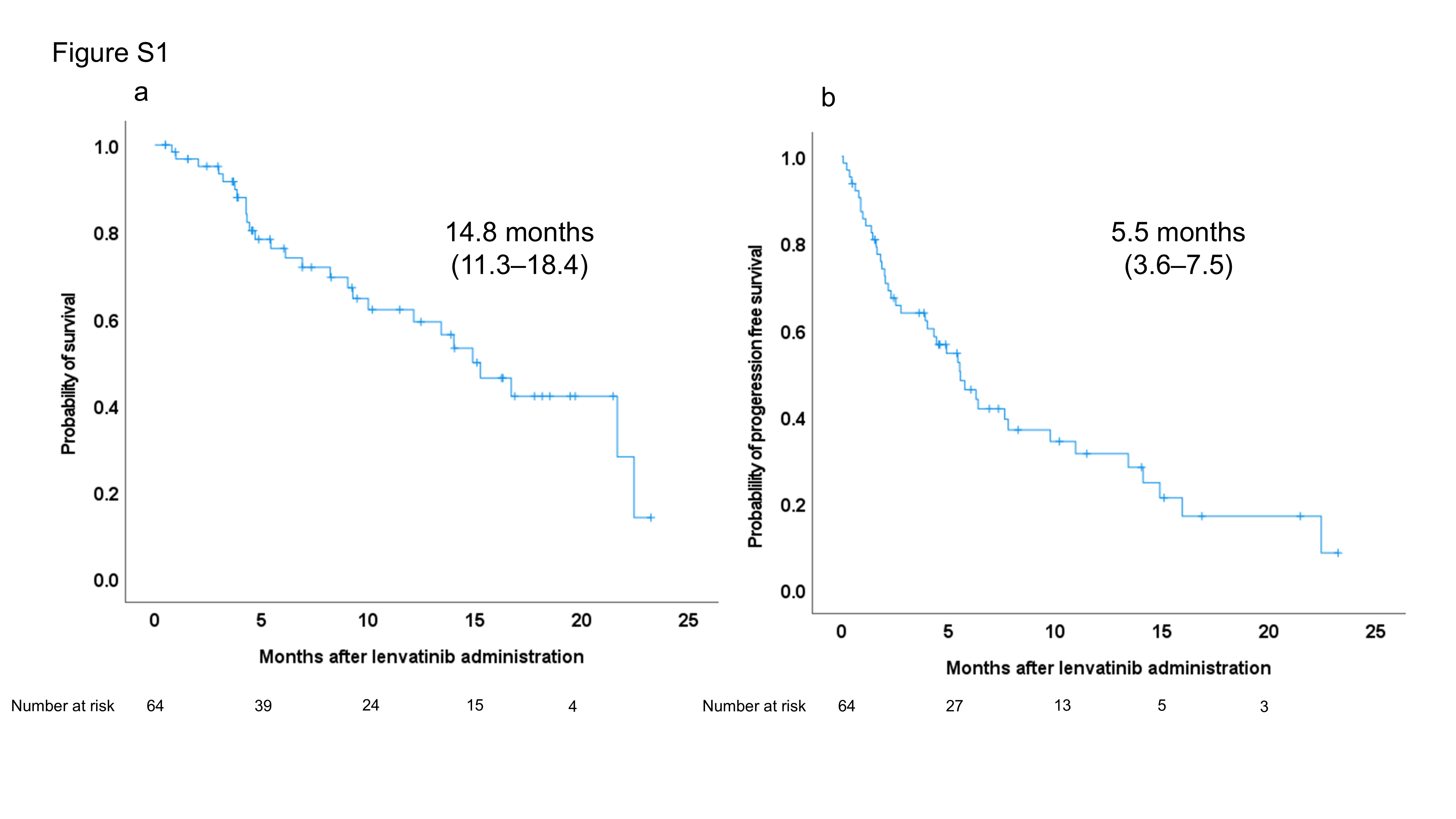

Supplement: Supplementary file 1 — FIGURE S1. (a) Median overall survival time after LEN was 14.8 months (95% confidence interval [CI], 11.3–18.4). (b) Median progression‐free survival time after LEN administration was 5.5 months (95% CI, 3.6–7.5). LEN, lenvatinib. [file JGH3-9-e70098-s002.tif]
