# Supplementary material for: Effect of Proteinuria Before Lenvatinib Administration on Treatment Response After Atezolizumab Bevacizumab Combination Therapy
Source: JGH Open. 2025 Jan 19;9(1):e70098. doi: 10.1002/jgh3.70098 (PMC11743979; doi:10.1002/jgh3.70098)
Supplement: Supplementary file 2 — TABLE S1. Baseline patient characteristics. [file JGH3-9-e70098-s004.docx]

Table S1. Baseline patient characteristics

| **Variable** | **N=64** |
| --- | --- |
| Sex  Male  Female | 48  16 |
| Age (years) | 74 (68–78) |
| Etiology  HCV  HBV  NBNC | 21  13  30 |
| ALBI score | -2.30 (-2.55–-1.94) |
| mALBI grade  1  2a  2b  3 | 12  21  26  5 |
| AFP (ng/mL) | 132 (10–2042) |
| BCLC stage  A  B  C | 2  22  40 |
| MVI | 18 |
| Metastasis | 26 |
| Duration of AB treatment (months) | 4.2 (1.3–7.8) |
| ORR of AB treatment (%) | 26.5 |
| Reason for stopping AB treatment  PD  AE  Others | 50  12  2 |
| UPCR | 0.51 (0.15–1.74) |
| qualitative value test　of urine protein  – or +-  +1  +2  +3≤ | 27  14  12  11 |
| Initial dose of lenvatinib  4 mg  8 mg  12 mg  Reduced dose | 6  44  14  22 |

Continuous data are presented as medians (25th–75th quartiles).

AB treatment, combination therapy of atezolizumab and bevacizumab; AE, adverse event; ALBI, albumin–bilirubin grade; mALBI, modified ALBI; AFP, alpha-fetoprotein; BCLC, Barcelona Clinic of Liver Cancer; HBV, hepatitis B virus; HCV, hepatitis C virus; MVI, major vascular invasion; metastasis, extrahepatic metastasis; NBNC, non-HBV, non-HCV; ORR, objective response rate; PD, progression disease; UPCR, urine protein/creatinine ratio
