# Supplementary material for: Effect of Proteinuria Before Lenvatinib Administration on Treatment Response After Atezolizumab Bevacizumab Combination Therapy
Source: JGH Open. 2025 Jan 19;9(1):e70098. doi: 10.1002/jgh3.70098 (PMC11743979; doi:10.1002/jgh3.70098)
Supplement: Supplementary file 3 — TABLE S2. Baseline patient characteristics in each group. [file JGH3-9-e70098-s001.docx]

Table S2. Baseline patient characteristics in each group.

| Variable | Group A (n=13) | Group B (n=51) | P-value |
| --- | --- | --- | --- |
| Sex  Male  Female | 10  3 | 38  13 | 1.00 |
| Age (years) | 77 (72–79) | 73(67–78) | 0.098 |
| Etiology  HCV  HBV  NBNC | 6  2  5 | 15  13  23 | 0.487 |
| Diabetes | 6 | 26 | 0.756 |
| Ischemic heart disease | 0 | 6 | 0.194 |
| ALBI score | -2.44 (-2.63–-2.17) | -2.21 (-2.52–-1.80) | 0.066 |
| mALBI grade  1  2a  2b  3 | 4  5  4  0 | 8  16  22  5 | 0.379 |
| AFP (ng/mL) | 135 (5–1693) | 129(13–2045) | 0.588 |
| BCLC stage  A  B  C | 1  7  5 | 1  15  35 | 0.125 |
| MVI | 1 | 17 | 0.089 |
| Metastasis | 5 | 23 | 0.761 |
| Duration of AB treatment (months) | 5.5(3.4–12.6) | 3.0(1.4–7.4) | 0.064 |
| Initial dose of lenvatinib  4 mg  8 mg  12 mg  Reduced dose | 2  6  5  3 | 4  38  9  19 | 0.143 |
| RDI | 53.0(33.1–68.9) | 57.9(36.0–87.1) | 0.258 |
| Use of weekend-off method | 4 | 8 | 0.301 |

Continuous data are presented as medians (25th–75th quartiles).

AB treatment, combination therapy of atezolizumab and bevacizumab; ALBI, albumin–bilirubin grade; mALBI, modified ALBI; AFP, alpha-fetoprotein; BCLC, Barcelona Clinic of Liver Cancer, MVI, major vascular invasion; metastasis, extrahepatic metastasis; RDI, relative dose intensity
