# Supplementary material for: Effect of Proteinuria Before Lenvatinib Administration on Treatment Response After Atezolizumab Bevacizumab Combination Therapy
Source: JGH Open. 2025 Jan 19;9(1):e70098. doi: 10.1002/jgh3.70098 (PMC11743979; doi:10.1002/jgh3.70098)
Supplement: Supplementary file 4 — TABLE S3. Treatment best response according to urinary protein level prior to lenvatinib administration. [file JGH3-9-e70098-s003.docx]

Table S3. Best response to treatment according to urinary protein level prior to lenvatinib administration.

|  | Group A | Group B |
| --- | --- | --- |
| CR | 0 | 1 |
| PR | 0 | 8 |
| SD | 5 | 24 |
| PD | 3 | 12 |
| NE | 5 | 6 |
| ORR | 0% | 17.6% |
| DCR | 38.4% | 64.7% |

CR, complete response; PR, partial response; SD, stable disease; PD, progression disease; NE, not examined; ORR, objective response rate; DCR, disease control rate.
